# New species of *Plectrocnemia* and *Nyctiophylax* (Trichoptera, Polycentropodidae) from China

**DOI:** 10.3897/zookeys.169.1827

**Published:** 2012-02-10

**Authors:** John C. Morse, Hua Zhong, Lian-fang Yang

**Affiliations:** 1 School of Agricultural, Forest, and Environmental Sciences, Clemson University, Clemson, SC, 29634-0310, USA; 2 Department of Entomology, Nanjing Agricultural University, Jiangsu, 210095, China

**Keywords:** Insecta, male genitalia, Oriental Biogeographic Region, East Palearctic Biogeographic Region, stream

## Abstract

Four new species of genus *Plectrocnemia* and 4 new species of genus *Nyctiophylax* are described, namely: *Plectrocnemia verticalis* **sp. n.**; *Plectrocnemia acuminata* **sp. n.**; *Plectrocnemia cryptoparamere* **sp. n.**; *Plectrocnemia qianshanensis* **sp. n.**; *Nyctiophylax (Nyctiophylax) senticosus* **sp. n.**; *Nyctiophylax (Paranyctiophylax) gracilis* **sp. n.**; *Nyctiophylax (Paranyctiophylax) pungens* **sp. n.**; and *Nyctiophylax (Paranyctiophylax) auriculatus* **sp. n.**

## Introduction

Dr Youwen Li included 4 new species of *Plectrocnemia* and 4 new species of *Nyctiophylax* in his doctoral dissertation (Li 1998) based on specimens collected by him, authors John C. Morse and Yang Lian-fang, and other colleagues during an expedition into southeastern and southcentral People’s Republic of China (PRC) in 1990. Although most of these species have been found many times in other Chinese localities since then, corroborating their standing as distinct species, their names have been unavailable until now. In addition to validating Li’s species, we provide here more detailed illustrations, descriptions, and distributions.

Since its original description, *Plectrocnemia* Stephens, 1836 (type species: *Plectrocnemia senex* Stephens, 1836 nec Pictet, 1834, monotypic, = *Plectrocnemia geniculata*, McLachlan 1871, according to Fischer 1962), has been reviewed by McLachlan (1878, for Europe), Ulmer (1907, for the world), Martynov (1934, for the former U.S.S.R.), Mosely (1939, for the United Kingdom), Tsuda (1942, for Japan), Mosely and Kimmins (1953, for Australia and New Zealand), and Ohkawa and Ito (2007, for Japan). The genus includes 97 extant species and 24 fossil species worldwide, with extant species occurring in the Oriental (48 spp.), West Palearctic (22 spp.), East Palearctic (19 spp.), and Australasian (8 spp.) Biogeographic Regions. The Chinese fauna presently includes 17 extant species. Four species described herein bring the total to 21 Chinese species of *Plectrocnemia*.

The genus *Nyctiophylax* Brauer, 1865 (type species: *Nyctiophylax sinensis* Brauer, monotypic) has been reviewed by Ulmer (1907, for the world), Morse (1972, for North America), and Neboiss (1993, for the world). *Paranyctiophylax* Tsuda, 1942 (type species: *Paranyctiophylax kisoensis* Tsuda, 1942, original designation) was redefined by Neboiss (1993) to include most of the species previously included in *Nyctiophylax*, but Malicky (1994), Li (1998), and Olah and Johanson (2010a, b) treated *Paranyctiophylax* as a subgenus of *Nyctiophylax*. The genus *Nyctiophylax* includes 107 extant species and 23 fossil species worldwide, with extant species occurring in the Oriental (58 spp.), Australasian (14 spp.), Afrotropical (12 spp.), Nearctic (10 spp.), East Palearctic (8 spp.), and Neotropical (5 spp.) Biogeographic Regions. The Chinese fauna presently includes 2 species. Four species described herein bring the total to 6 Chinese species of *Nyctiophylax*.

The monophyly of each of these genera and the relationships of the genera of Polycentropodidae were recently explored according to morphological characters by Chamorro and Holzenthal (2011).

## Methods

Adults were collected with ultraviolet lights unless otherwise indicated. The abdomens of males were cleared with a heated KOH solution to reveal internal and other hidden structures. Each dissected abdomen is preserved with the remainder of its specimen in 80% ethyl alcohol.

Pencil drawings were prepared through use of an ocular grid in a Wild® M5 dissecting microscope, then these pencil templates were inked with various sizes of Rapidograph® pens. In the descriptions, colors are those observed for the specimens in alcohol. The morphological terms for male genitalia and wing venation follow Arefina et al. (2003), Ohkawa and Ito (2007), and Hamilton and Holzenthal (2011). All specimens, except as noted, have been deposited in the Nanjing Agricultural University Insect Collection (NAU) and the Clemson University Arthropod Collection (CUAC).

## Species descriptions

### Plectrocnemia verticalis

Morse, Zhong & Yang sp. n.

urn:lsid:zoobank.org:act:606DA6D6-74FA-4282-92F1-692E79B9139B

http://species-id.net/wiki/Plectrocnemia_verticalis

Fig. 1

Plectrocnemia verticalis Li 1998: 51–52, figs 3.7–3.9, *nomen nudum*.

#### Type material.

Holotype male, PRC, Yun-nan Province, Ji-ping County, Kun-he Village, 10 km N of A-de-bo Township, 22.80°N, 103.30°E, 1350 m elevation, 19-vii-1990, collected by Li You-wen, Ke Xin; deposited in NAU.

#### Diagnosis.

The male genitalia of the species are somewhat similar to those of *Plectrocnemia banksi* Fischer, 1962, in that each preanal appendage has a dorsal process and the inferior appendages are concave on their subapicomesal margins. However, the preanal appendages are somewhat crescentic in lateral view and the apex of the lower arm of each preanal appendage is acute with a hairy wart on the ventral margin near the middle in the new species, characters which are unique in the genus.

#### Description of adult male:

Color of unique (holotype) specimen in alcohol generally yellowish brown. Length of body with folded wings: 7.0 mm.

**Figure 1.**
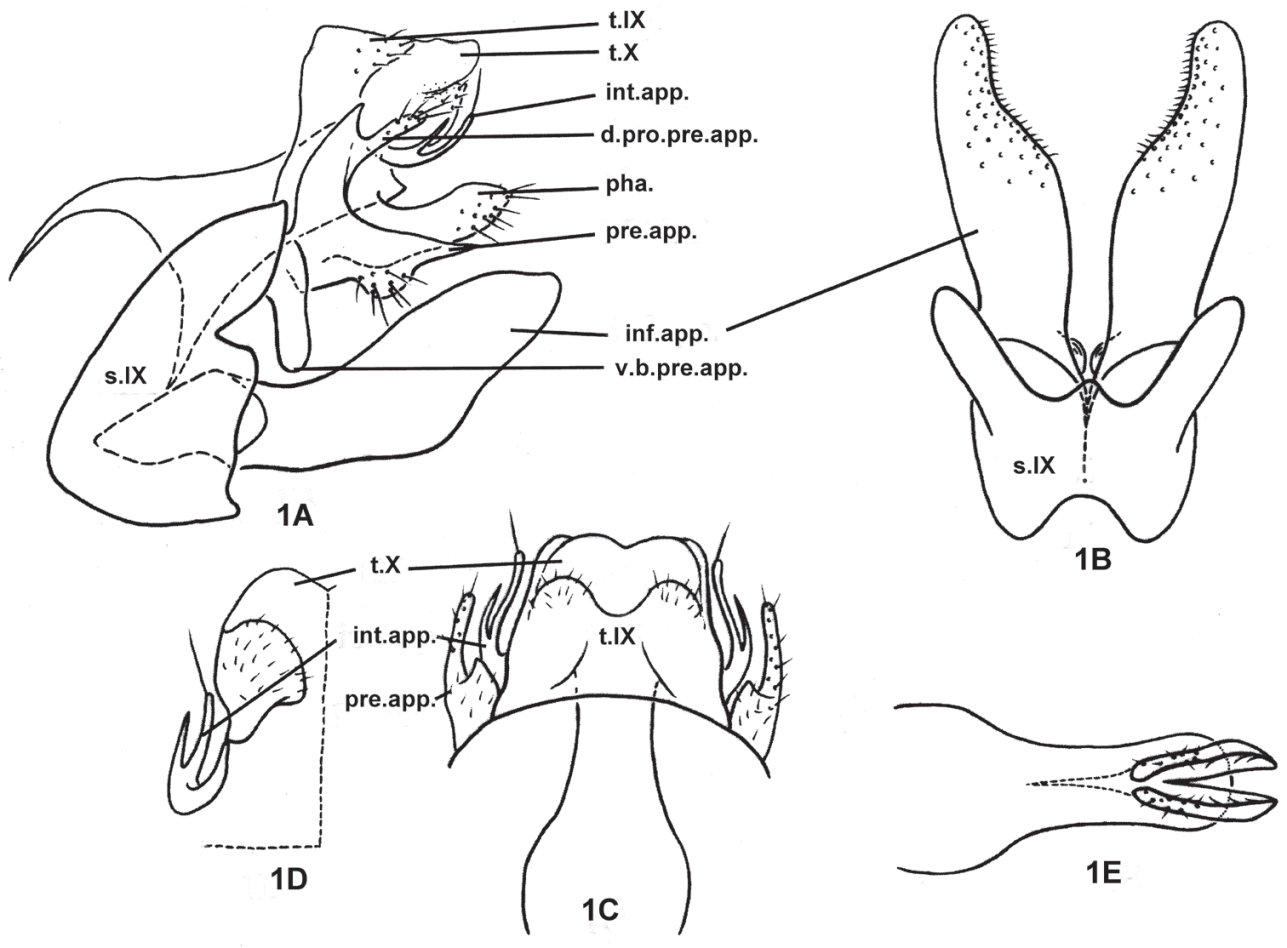
*Plectrocnemia verticalis* Morse, Zhong & Yang, sp. n., male genitalia. **1A** left lateral view **1B** ventral view **1C** dorsal view **1D** tergum X, ventral view **1E** phallus, ventral view. d.pro.pre.app. = dorsal process of a preanal appendage; inf.app. = inferior appendage; int.app. = intermediate appendage; pha. = phallus; pre.app. = preanal appendage; s.IX = sternum IX; t.IX = tergum IX; t.X = tergum X; v.b.pre.app. = ventrobasal branch of preanal appendage.

Male genitalia: Tergum IX semi-membranous, with pair of setose patches posteriorly; sternum IX with anteromesal margin convex and posteromesal margin deeply excised except for small, median, triangular protrusion (Fig. 1B), posterolateral margins each with triangular incision at 1/3rd distance from dorsal margin (Fig. 1A). Tergum X semi-sclerotized, broadly trapezoidal, and with base covered by tergum IX in dorsal view (Fig. 1C). Intermediate appendages, set basolaterally from X, each slender, curved posterodorsad, forked in distal half, with longer ventral branch bearing long, apical seta (Figs 1A, 1C, 1D). Preanal appendages somewhat crescentic in lateral view (Fig. 1A); each with slender, setose dorsal process directed posterad and shorter than tergum X; lower branch longer than tergum X and shorter than inferior appendages, with hairy wart on ventral margin near middle and with apex acute; ventrobasal branch of each preanal appendage vertical, thick, and thumb-like. Subphallic sclerite absent. Inferior appendages sclerotized, each compressed, broadly foliaceous, tapering from middle to apex in lateral view (Fig. 1A); in ventral view (Fig. 1B) densely covered with short, stout setae and concave mesally on distal 1/3rd, each with small mound basomesally. Phallus (Fig. 1E) tube-like, with pair of lanciform projections arising subapicoventrally and exceeding apex of phallic tube, each with row of ventral hairs; without paramere spines or internal phallic sclerites..

#### Female and immature stages.

Unknown.

#### Etymology:

*Verticalis*, Latin adjective, “vertical,” referring to the vertical ventrobasal branch of each preanal appendage.

#### Distribution.

Oriental Biogeographic Region, China (Yun-nan). The species, known only from the holotype, has been found only at the type location in southern Yun-nan Province.

### Plectrocnemia acuminata

Morse, Zhong & Yang sp. n.

urn:lsid:zoobank.org:act:35C1A91C-B319-4B05-AAF6-7D12FA4C245C

http://species-id.net/wiki/Plectrocnemia_acuminata

Fig. 2

Plectrocnemia acuminata Li 1998: 54–55, figs 3.13–3.15, *nomen nudum*.

#### Type specimens.

Holotype male, PRC, Si-chuan Province, Mt. Qing-cheng, Wei-jiang River, 32 km SW of Guan County (now, Du-jiang-yan City), 31.00°N, 103.60°E, 930 m elevation, 20-vi-1990, collected by JC Morse, Yang Lian-fang, Li You-wen; deposited in NAU.

Paratypes: PRC, Si-chuan Province: same data as hololype, 3 males (CUAC); Ya-an, Zhou-gong Stream branches, alt. 600–800 m elevation, 08-vi-1996, coll. Wang Bei-xin, 9 males (NAU); Mei-gu County, Mei-gu Da-feng-ding National Nature Preserve, Shu-wo-xiang village, Cha-cha-ku Stream, 8.3 km E of Long-wo, 28.7603°N, 103.2471°E, 1671 m elevation, 06-vii-2005, Coll. Sun Chang-hai, 1 male (NAU).

PRC, An-hui Province: Yang-jia-tan, Feng-yuan-shui Stream, Xi-xian County, 29.90°N, 118.45°E, 215 m elevation, 24-v-1992, collected by JC Morse, Sun Chang-hai, 4 males (NAU).

#### Diagnosis.

The male genitalia of this species are very similar to those of *Plectrocnemia munitalis* Mey, 1996 in the apically acute preanal appendages and in the presence of a hooked basodorsal process on each inferior appendage. However, the intermediate appendages of segment X are slender, longer than the inferior appendages in *Plectrocnemia munitalis*, but much shorter, and forming irregular, broad plates each with an acute posterior projection directed dorsolaterad in the new species.

#### Description of adult male:

Head and thorax dark brown with antennae and warts yellowish brown, forewings light brown. Length of body with folded wings: 7.0 – 9.0 mm. (n = 10).

**Figure 2.**
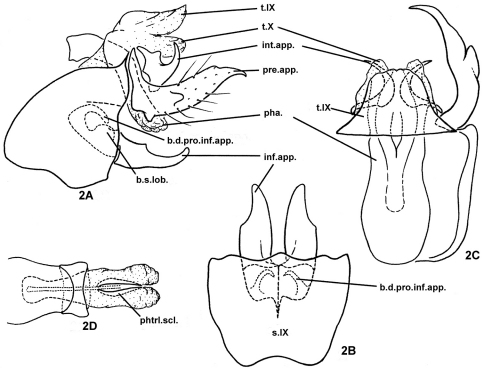
*Plectrocnemia acuminata* Morse, Zhong & Yang, sp. n., male genitalia. **2A** left lateral view **2B** ventral view **2C** dorsal view **2D** phallus, dorsal view. b.d.pro.inf.app. = basodorsal process of an inferior appendage; b.s.lob. = basomesal setose lobe of an inferior appendage; inf.app. = inferior appendage; int.app. = intermediate appendage; pha. = phallus; phtrl. scl. = phallotremal sclerite; pre.app. = preanal appendage; s.IX = sternum IX; t.IX = tergum IX; t.X = tergum X.

Male genitalia. Highly sclerotized. Tergum IX fused basally with broader tergum X, with posterior margin narrower than anterior margin in dorsal view (Fig. 2C); sternum IX with anteromesal margin concave (Fig. 2B), anterolateral margins broadly protruding cephalad at middle, posterolateral margins sinuate (Fig. 2A). Intermediate appendages each short, irregularly broad plate beneath and beside tergum X, with acute posterior projection directed dorsolaterad (Figs 2A, 2C). Preanal appendages nearly twice as long as tergum X and inferior appendages, each cylindrical, with distal portion narrowing more or less evenly to acute apex, evenly curved mesad, with small conical ventrobasal projection and with short lobe projecting posterolaterad from its lateral surface near middle; ventral subphallic sclerite absent (Figs 2A, 2C). Inferior appendages depressed (flattened dorsoventrally), each evenly broad at basal half with lateral margin of distal half shallowly excised (Fig. 2B); basodorsal process of each inferior appendage slender, hooked dorsomesad (Figs 2B, 2A); basal setose lobe on dorsal base of each inferior appendage rounded (Fig. 2A), both basodorsal process and basal setose lobe hidden within segment IX. Phallus with long and broad phallobase somewhat constricted subapically; sclerotized ring near middle of phallus about 1/4th as long as phallobase; phallicata constricted subbasally, membranous apically, with pair of thin, short phallotremal sclerites visible from dorsal view (Fig. 2D).

#### Female and immature stages.

Unknown.

#### Etymology.

*Acuminata*, Latin adjective, “narrowed,” referring to the distal portion of each preanal appendage gradually narrowing to an acute apex.

#### Distribution.

Oriental Biogeographic Region, China (Si-chuan, An-hui).

### Plectrocnemia cryptoparamere

Morse, Zhong & Yang sp. n.

urn:lsid:zoobank.org:act:83B082DB-74DA-4405-9F3D-2D24238037AA

http://species-id.net/wiki/Plectrocnemia_cryptoparamere

Fig. 3

Plectrocnemia cryptoparamere Li 1998: 67–68, figs 3.52–3.55, *nomen nudum*.

#### Type material.

Holotype male, PRC, Hu-bei Province, Ma-cheng County, Tong-jian-chong River, 27 km N. of Ma-cheng, 31.10°N, 115.01°E, 12-vii-1990, 150 m elevation, collected by JC Morse & Yang Lian-fang; deposited in NAU.

#### Paratypes.

PRC, Hu-bei Province: same data as holotype, 10 males, deposited in NAU (6 males) and CUAC (4 males).

PRC, Jiang-xi Province: Mt. Wu-yi National Nature Preserve, Unnamed tributary of Tong-Mu River, 18 km upstream of Mt. Wu-Yi Station, 27.8275°N, 117.74356°E, 1450 m elevation, 02-vi-2005, Coll. Sun Chang-hai, 1 male (NAU).

PRC, Guang-dong Province: Zhao-qing City, Ding-hu District, Mt. Ding-hu Forest Ecosystem Research Station, Academia Sinica, Dong Gou at Shui-lian-dong-tian waterfall, 23.1604°N, 112.5250°E, 170 m elevation, 24-v-2004, Coll. CJ Geraci, JC Morse, Sun Chang-hai, 3 males (NAU).

#### Diagnosis.

The male genitalia of this species are similar to those of *Plectrocnemia plicata* Schmid, 1959 and *Plectrocnemia wui* (Ulmer, 1932) in the shape of the posterolateral margins of sternum IX with a conspicuous division at its middle on each side in lateral view, inferior appendages broad and truncate in lateral view, and preanal appendages each with a long, needle-like mesoventral process. However, the new species has acute posterior dorsolateral margins on sternum IX (these margins are blunt and rectilinear in the other 2 species), narrow and parallel-sided preanal appendages in lateral view (oval in the other 2 species), the ventromesal process of each inferior appendage is triangular in ventral view and broadly connected basally with the body of the appendage (digitate and deeply separated from the body of the appendage in the other 2 species), and it lacks parameres on the phallus (parameres are present in the other 2 species).

#### Description of adult male.

Head brown with antennae and palpi pale yellow, pronotum light brown, meso- and metanota brown with yellowish warts, forewings light brown. Length of body with folded wings: 6.8 – 7.2 mm. (n = 5).

**Figure 3.**
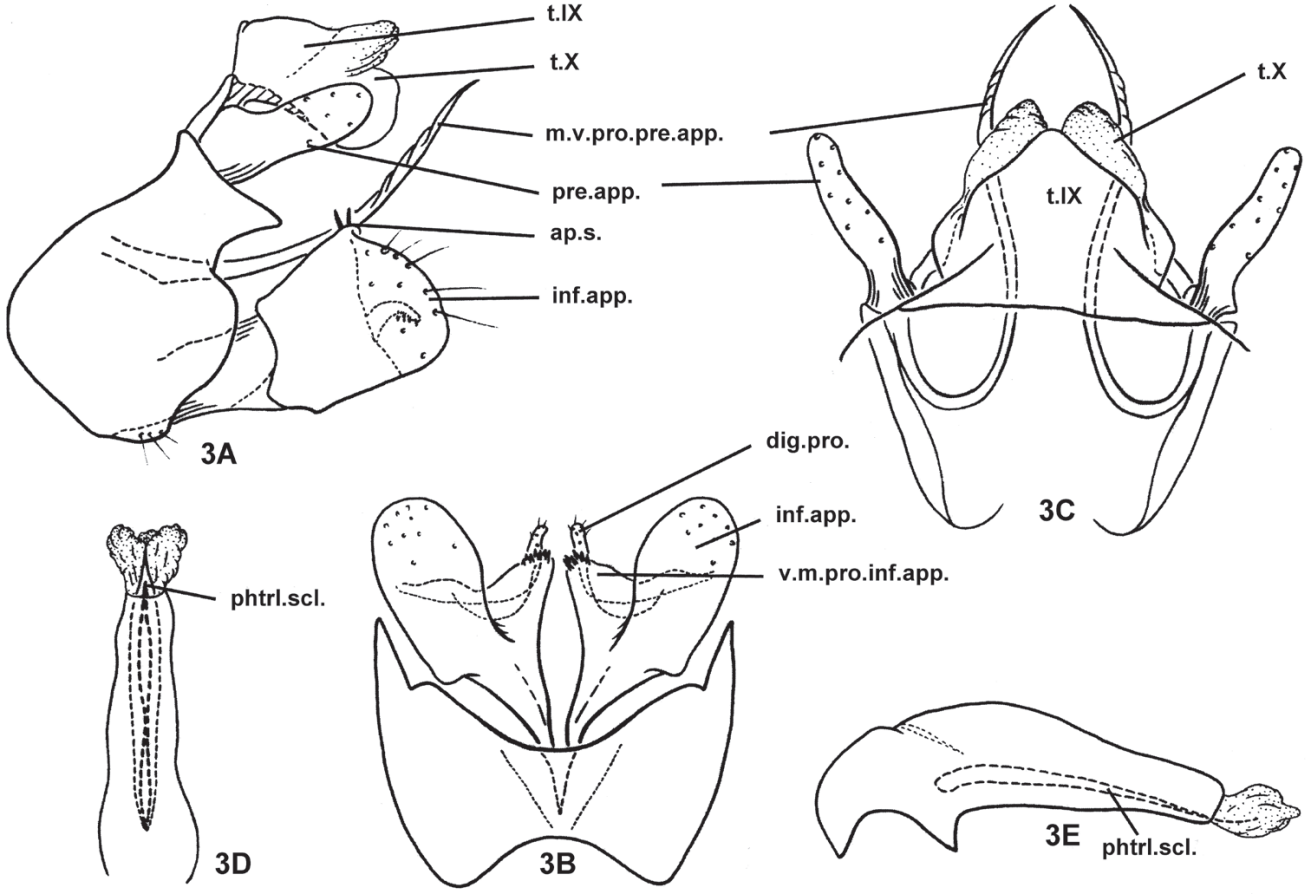
*Plectrocnemia cryptoparamere* Morse, Zhong & Yang, sp. n., male genitalia. **3A** left lateral view **3B** ventral view **3C** dorsal view **3D** phallus, ventral view **3E** phallus, left lateral view. ap.s. = apical setae of mesal plate of an inferior appendage; dig.pro. = digitate process of a mesal plate of an inferior appendage; inf.app. = inferior appendage; m.v.pro.pre.app. = mesoventral process of a preanal appendage; phtrl.scl. = phallotremal sclerite; pre.app. = preanal appendage; s.IX = sternum IX; t.IX = tergum IX; t.X = tergum X; v.m.pro.inf.app. = ventromesal process of an inferior appendage.

Male genitalia. Tergum IX fused with tergum X; upper lobes of tergum X semi-membranous, lower lobes of tergum X sclerotized, both upper and lower lobes divided apicomesally, each lobe with rounded apex (Figs 3A, 3C). In lateral view (Fig. 3A), sternum IX narrowed subdorsally, its anterior margins broadly protruding cephalad at middle, posterior margins sinuate, with posterior dorsolateral corners produced in acute, triangular process on each side; anteromesal and posteromesal margins concave, lower posterolateral margins broadly convex (Fig. 3B). Intermediate appendages not well developed, represented only as thickened lateral margins of tergum X, slightly broader at base, narrowing to acute apex. Preanal appendages slightly shorter than tergum X, each broader at base, narrower and straight from basal 1/3rd to rounded apex (Figs 3A, 3C); mesoventral process of each preanal appendage recurved anterad then posterad, needle-like, wrinkled distally, guided by semi-membranous string across genitalia chamber between bases of inferior appendages, this string represented in other species by sclerotized subphallic sclerite. Inferior appendages compressed (flattened from side to side), subquadrate, about as long as tall, with distal margin broad and truncate in lateral view (Fig. 3A), distinctive mesal plate forming short vertical ridge with 2–3 short apicodorsal setae in lateral view (3A); in ventral view (Fig. 3B) ventromesal processes of inferior appendages each triangular with blunt apex covered with tiny teeth; basal digitate process of each inferior appendage mesal plate slender and simple, conspicuously extending beyond mesal plate. Phallus broad at base, more slender and tapered distally, without parameres, with pair of slender phallic sclerites (possibly elongated phallotremal sclerite) visible in ventral and lateral views (Figs 3D, 3E).

#### Female and immature stages.

Unknown.

#### Etymology.

*Crypt*, Greek adjective, “hidden,” referring to the absence of phallic parameres.

#### Distribution.

Oriental Biogeographic Region, China (Hu-bei, Jiang-xi, Guang-dong).

### Plectrocnemia qianshanensis

Morse, Zhong & Yang sp. n.

urn:lsid:zoobank.org:act:BFF78E8A-CBF8-4513-8846-5A63FCC47BB8

http://species-id.net/wiki/Plectrocnemia_qianshanensis

Fig. 4

Plectrocnemia qianshanensis Li 1998: 68–69, figs 3.56–3.59, *nomen nudum*.

#### Type material.

Holotype male, PRC, Jiang-xi Province, Qian-shan County, Shi-long, 27.20°N, 114.08°E, 24-vii-1993, collected by Lu Liang; deposited in NAU.

#### Paratypes.

PRC, Jiang-xi Province: Mt. Jiu-lian National Nature Preserve, confluence of Huang-niu-shi & Da-shui-keng Streams, 1.2 km SE of Dun-tou Village, 24.5256°N, 114.4225°E, 546 m elevation, 09-vi-2005, Coll. Sun Chang-hai, 2 males (NAU).

PRC, An-hui Province: Qi-men County, 29.8°N, 117.7°E, Peng-long Township, Yang Village, 122 km on Provincial Road, 25-viii-2002, Coll. Hu Ben-jin, Lu Shuang, 1 male (NAU).

PRC, Shaan-xi Province: Fu-ping County, Long-cao-ping Village, 1100 m elevation, 03-vi-1998, coll. Sun Chang-hai, 1 male (NAU).

#### Diagnosis.

The male genitalia of this species are similar to those of *Plectrocnemia plicata* and *Plectrocnemia wui* in the shape of the posterolateral margins of sternum IX, with a conspicuous division at the middle, in the inferior appendages being broad and truncate in lateral view. However, in this new species, although the preanal appendages are narrow and parallel-sided as in *Plectrocnemia cryptoparamere*, these appendages are distinctly shorter than tergum X (subequal in the other 3 species). The posterior dorsolateral corners of sternum IX are rounded on each side in this species (blunt and rectilinear in *Plectrocnemia plicata* and *Plectrocnemia wui*, acute in *Plectrocnemia cryptoparamere*). A subphallic sclerite is present in this new species (absent in the other 3 species). Like *Plectrocnemia cryptoparamere*, the ventromesal process of each inferior appendage is triangular in ventral view and broadly connected basally with the body of the appendage (digitate and deeply separated from the body of the appendage in *Plectrocnemia plicata* and *Plectrocnemia wui*), but the main body of each inferior appendage is apically truncate in ventral view in this species (rounded in the other 3 species). As in *Plectrocnemia plicata* and *Plectrocnemia wui*, the phallus of this species bears paramere spines (parameres are lacking in *Plectrocnemia cryptoparamere*). The new species has the mesoventral process of each preanal appendage forked (unforked in the other 3 species).

#### Description of adult male.

Head brown with antennae and palpi pale yellow, pronotum light brown, meso- and metanota brown with yellowish warts, forewings greyish brown. Length of body with folded wings: 6.0–6.5 mm. (n=4).

**Figure 4.**
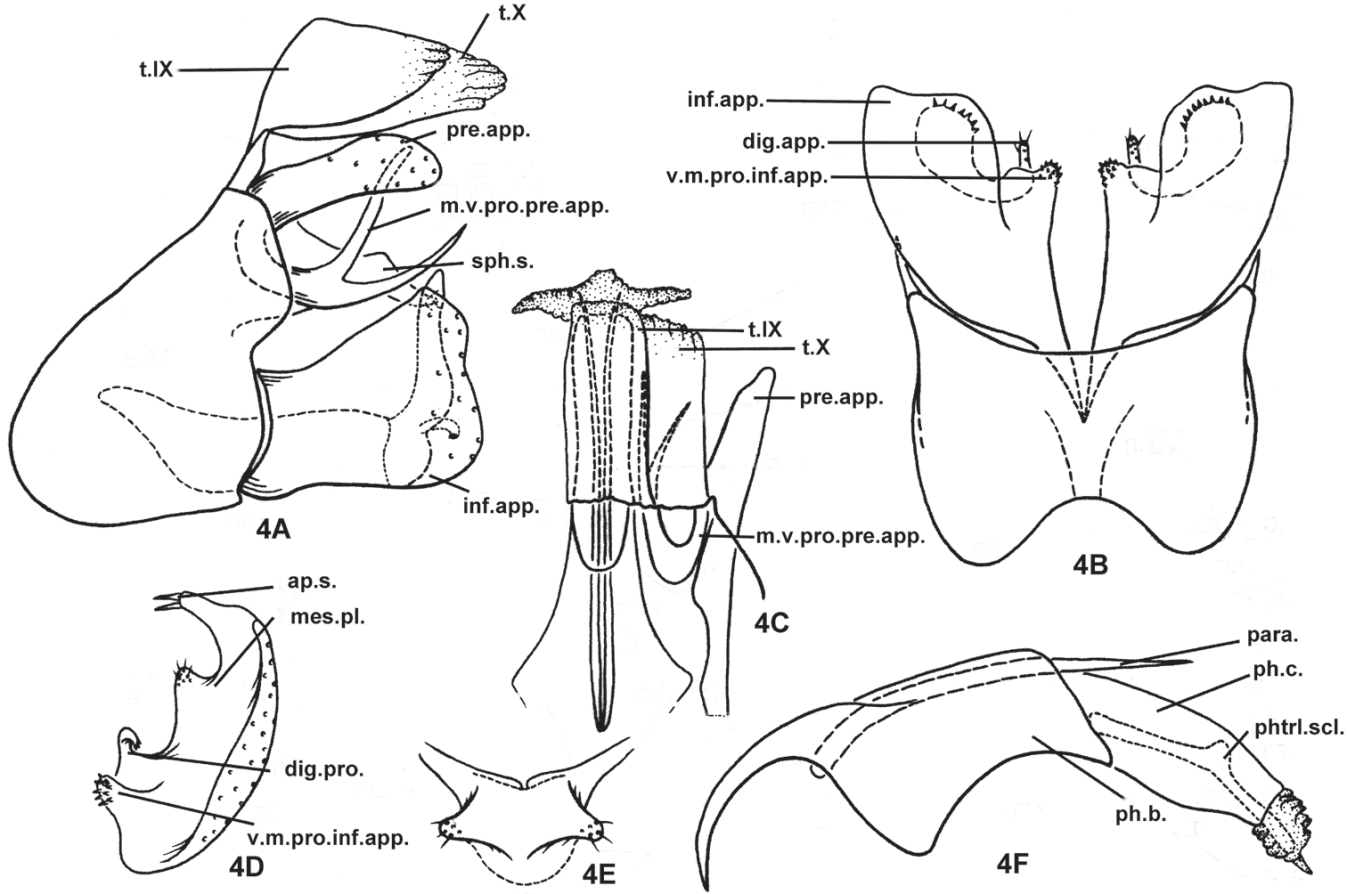
*Plectrocnemia qianshanensis* Morse, Zhong & Yang, sp. n., male genitalia. **4A** left lateral view **4B** ventral view **4C** dorsal view **4D** right inferior appendage, caudal view **4E** subphallic sclerite, caudal view **4F** phallus, left lateral view. ap.s. = apical setae of mesal plate of an inferior appendage; dig.pro. = digitate process of a mesal plate of an inferior appendage; inf.app. = inferior appendage; mes.pl. = mesal plate of an inferior appendage; m.v.pro.pre.app. = mesoventral process of a preanal appendage; para. = paramere; ph.b. = phallobase; ph.c. = phallicata; phtrl. scl. = phallotremal sclerite; pre.app. = preanal appendage; s.IX = sternum IX; sph.s. = subphallic sclerite; t.IX = tergum IX; t.X = tergum X; v.m.pro.inf.app. = ventromesal process of an inferior appendage.

Male genitalia. Tergum IX semimembranous apically, fused with semimembranous tergum X (t.X), these fused terga as long as inferior appendages (Figs 4A, 4C). In ventral view (Fig. 4B) anteromesal margin of sternum IX narrowly excised, posteromesal margin broadly excised; in lateral view (Fig. 4A) sternum IX subtriangular, anterior margin broadly rounded and projecting anterad; posterior margin sinuate, with upper half protruding posterad beyond lower half. Intermediate appendages absent. Preanal appendages slightly shorter than fused terga IX and X, about 4 times as long as wide in lateral view (Fig. 4A), each parallel-sided, with rounded apex; mesoventral process of each preanal appendage heavily sclerotized, divided into upper and lower branches at middle and both curved dorsad, with upper branch nearly vertical and apically blunt in lateral view, lower branch slightly more slender and acute, and exceeding slightly beyond apex of preanal appendages; subphallic sclerite long, its paired apices almost reaching tips of inferior appendages in lateral view, in caudal view (Fig. 4E) united basomesally with each other, each with setose, thumb-like apex directed caudolaterad. Inferior appendages each about 1.5 times as long as its mid width, narrowed at base and with distal margin broad and sinuously truncate in lateral view (Fig. 4A); in ventral view (Fig. 4B) ventromesal process of each inferior appendage quadrate, with mesal end covered with tiny teeth (Figs 4B, 4D); vertical mesal plate with basal digitate process (dig.pro.) conspicuous, slender, simple, hooked laterad; in caudal view (Fig. 4D) mesal plate well developed with 2 stout apicodorsal setae on elongate process, and additionally with 1 short, blunt, setose lobe between elongate process and basal digitate process. Phallus with broad phallobase, more-slender and parallel-sided phallicata, pair of long, paramere spines (para.); pair of phallotremal sclerites (or phallic sclerites) slender, about as long as phallicata (Fig. 4F).

#### Female and immature stages.

Unknown.

#### Etymology.

The species is named after the holotype locality.

#### Distribution.

East Palearctic and Oriental Biogeographic Regions, China (Jiang-xi, An-hui, Shaan-xi).

### Nyctiophylax (Nyctiophylax) senticosus

Morse, Zhong & Yang sp. n.

urn:lsid:zoobank.org:act:5840A196-3704-443F-BC23-8228D4140E25

http://species-id.net/wiki/Nyctiophylax_senticosus

Fig. 5

Nyctiophylax (Nyctiophylax) senticosus Li 1998: 89–90, figs 4.1–4.3, 4.9–4.12, *nomen nudum*.

#### Type materials.

Holotype male, PRC, An-hui Province, Jin County, Song Village, Ding-xi Stream, 33 km E. of Jin County, 30.70°N, 118.35°E, 08-vi-1990, 120 m elevation, collected by JC Morse, Sun Chang-hai, Yang Lian-fang; deposited in NAU.

#### Paratypes.

PRC, An-hui Province: same data as holotype, 4 males (NAU).

PRC, Guang-xi Province: Shang-si County, Na-lin Stream, tributary of Ming-jiang River, 2.0 km NW of main entrance to Mt. Shi-wan-da National Forest Park, 21.9070°N, 107.8966°E, 281 m elevation, 05-vi-2004, Coll. JC Morse, Sun Chang-hai, 3 males (NAU); Shang-si County, Na-lin Stream, tributary of Ming-jiang River, 2.2 km NW of main entrance to Mt. Shi-wan-da National Forest Park, 21.9062°N, 107.8962°E, 284 m elevation, 05-vi-2004, Coll. Zhou Xin, Karl M Kjer, 2 males (NAU); Shang-si County, Mt. Shi-wan-da National Forest Park, Shi-tou Stream, tributary of Ming-jiang River, 1.35 km SW of main entrance to Park, 21.9022°N, 107.9046°E, 300 m elevation, 05-vi-2004, Coll. Yang Lian-fang, CJ Geraci, 4 males (NAU); Xing-an County, Liu-dong Stream and Hua-jiang Stream confluence, ~1 km S of Hua-jiang town, 25.7657°N, 110.4820°E, 262 m elevation, 16-vi-2004, Coll. Yang Lian-fang, JC Morse, Sun Chang-hai, CJ Geraci, 4 males (NAU).

#### Diagnosis.

The male genitalia of *Nyctiophylax (Nyctiophylax) senticosus* are somewhat similar to those of *Nyctiophylax (Nyctiophylax) sinensis* Brauer, 1865 in the deeply inserted dorsal processes of the preanal appendages set under tergum VIII, but differ from those of *Nyctiophylax (Nyctiophylax) sinensis* by the inferior appendages with their acute apices strongly curved dorsad in the new species; they are almost straight in lateral view with the apices rounded in *Nyctiophylax sinensis*.

The male genitalia of *Nyctiophylax (Nyctiophylax) senticosus* are also very similar to those of *Nyctiophylax (Nyctiophylax) maath* (Malicky & Chantaramongkol, 1993) in the following characters: 1) The preanal appendages have a ventral lobe; 2) the preanal appendages have their dorsal processes inserted under tergum VIII; and 3) the inferior appendages have basomesal processes. However, the differences between the 2 species are obvious. The apex of each inferior appendage of *Nyctiophylax maath* is straight, like that of *Nyctiophylax sinensis*, while it is up-curved in the new species. The basomesal processes of the inferior appendages in *Nyctiophylax maath* are hook-like, mostly exposed in ventral view, but in the new species club-like, densely covered with tiny teeth apically, and with only the apices exposed.

#### Description of adult male.

Head grayish brown with yellowish antennae, pronotum light brown, meso- and metanota brown, with thoracic legs yellowish, forewings light brown. Length of body with folded forewings: 4.5–4.8 mm (n=10).

**Figure 5.**
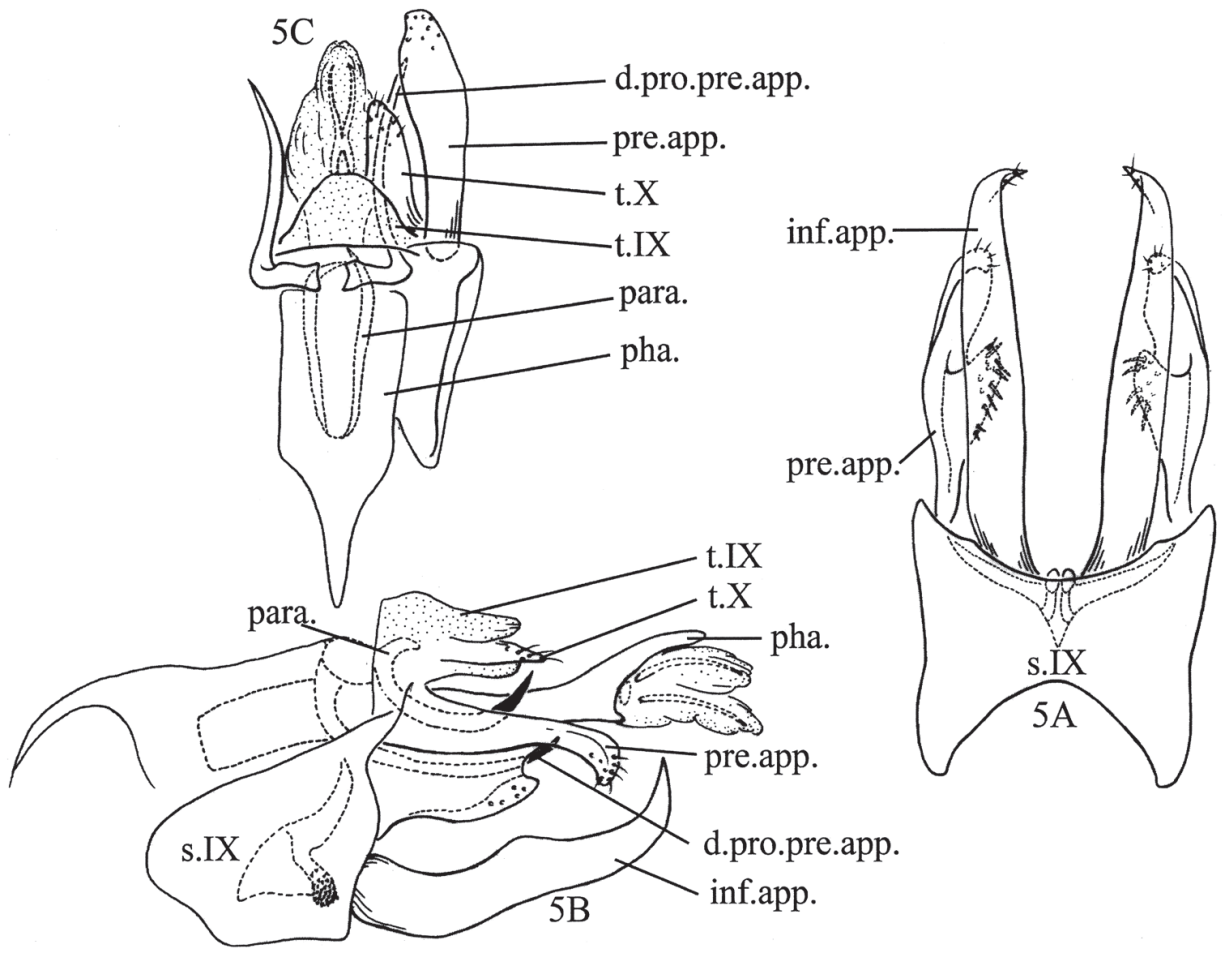
*Nyctiophylax (Nyctiophylax) senticosus* Morse, Zhong & Yang, sp. n., male genitalia. **5A** left lateral view **5B** ventral view **5C** dorsal view. d.pro.pre.app. = dorsal process of a preanal appendage; inf.app. = inferior appendage; para. = paramere; pha. = phallus; pre.app. = preanal appendage; s.IX = sternum IX; t.IX = tergum IX; t.X = tergum X.

Male genitalia. Tergum IX parabolic in dorsal view (Fig. 5C), membranous, almost transparent. In lateral view (Fig. 5A), sternum IX obliquely protruded anterad subventrally, posterolateral margins vertical and sinuous, ventral surface horizontal and as long as anteroventral margins; in ventral view (Fig. 5B), posterior and anterior margins each with wide, shallow excision. Tergum X semi-sclerotized, setose, deeply divided apicomesally (Figs 5A, 5C).Preanal appendages slightly longer than tergum X, each with broad lobe along basal 2/3rds of ventral edge (Fig. 5A), this lobe with setose basoventral apex directed ventromesad in ventral view (Fig. 5B); dorsal processes of preanal appendages inserted deeply under tergum VIII, each consisting of broad basal plate with long, slender process coiled laterad, ventrad, and then caudad to acute, out-turned apex. Inferior appendages depressed (flattened dorsoventrally), about 1.5 times as long as sternum IX, each with acute apex curved dorsad; with short, club-like, basomesal projection hidden inside sternum IX, its apex densely covered with tiny teeth. Phallus with thick phallobase half as long as entire phallus; pair of parameres curved dorsad; phallicata membranous, enlarged to apex, with 2 spines among retracted membranes.

#### Female and immature stages.

Unknown.

#### Distribution.

Oriental Biogeograpic Region, China (An-hui, Guang-xi).

#### Etymology.

*Senticosus*, Latin, „of many spines,“ referring to the spines of the phallus.

### Nyctiophylax (Paranyctiophylax) gracilis

Morse, Zhong & Yang sp. n.

urn:lsid:zoobank.org:act:AD32B3CC-FE44-41F4-8DD5-4AB7D3B06785

http://species-id.net/wiki/Nyctiophylax_gracilis

Fig. 6

Nyctiophylax (Paranyctiophylax) gracilis Li 1998: 92–93, figs 4.13–4.18, *nomen nudum*.

#### Type material.

Holotype male, PRC, Jiang-xi Province, 38 km. from Cong-an City and 2 km from Jiang-xi Provincial border at 80-km marker, 27.78°N, 118.03°E, 550 m elevation, 26-v-1990, collected by Sun Chang-hai; deposited in NAU.

#### Paratypes.

PRC, Jiang-xi Province: same data as holotype, 11 males (NAU); Mt. Jiu-lian National Nature Preserve, unnamed tributary 0.5 km from Xia-Gong-Tang Stream, 24.5347°N, 114.4689°E, 630 m elevation, 07-vi-2005, Coll. Zhou Xin, Sun Chang-hai, 5 males (NAU).

PRC, Zhe-jiang Province: Mt. Tian-mu, 30.4°N, 119.5°E, San-mu-ping, 780 m elevation, 26–29-vii-1998, black light, coll. Wu Hong, 8 males (NAU); San-mu-ping, 780 m elevation, 23-vi-1998, black light, coll. Zhao Ming-shui, 4 males (NAU); Kai-shan-lao-dian, 1090 m elevation, 14vii-1999, light, coll. Zhao Ming-shui, 2 males (NAU); San-mu-ping, 780 elevation, 15-ix-1998, coll. Zhao Ming-shui, 1 male (NAU); San-mu-ping, 780 m elevation, 01-vii-1998, coll. Wu Hong, 1 male (NAU); An-ji County, 30.6345°N, 119.676°E, Mt. Long-wang, 400 m elevation, 03-vi-1999, Coll. Du Yu-zhou, 2 males (NAU).

PRC, An-hui Province: Qi-men County, 50 m upstream of Shu-ang-he-kou, Tang-yun-li tributary, 29.8°N, 117.7°E, 530 m elevation, 30-v-2002, coll. Shan Lin-na, Hu Ben-jin, 2 males (NAU); Shu-ang-he-kou, Tao-yuan-li tributary, 29-ix-2003; coll. Shan Lin-na, Sun Chang-hai, 2 males (NAU); Shu-ang-he-kou, Tao-yuan-li tributary, 26-viii-2003, coll. Sun Chang-hai, Shan Lin-na, 17 males (NAU).

PRC, Guang-xi Province: Shang-si County, Mt. Shi-wan-da National Forest Park, 1st tributary of Shi-tou Stream, Zhu-jiang-yuan Waterfall, ~4 km SW of main entrance to Park, 485 m elevation, 06-vi-2004, Coll. Zhou Xin, Karl M Kjer, 1 male (NAU).

PRC, Si-chuan Province: Mei-gu County, Mei-gu Da-feng-ding National Nature Preserve, Shu-wo Village, Cha-cha-kou Stream, 9.0 km E of Long-wo, 28.7608°N, 103.2535°E, 1650 m elevation, 06-vii-2005, Coll. CJ Geraci, JC Morse, 4 males (NAU); Mei-gu County, Mei-gu Da-feng-ding National Nature Preserve, Shu-wo Village, Gong-fan-yi Stream, 9.5 km E of Long-wo, 28.7605°N, 103.2581°E, 1653 m elevation, 06-vii-2005, Coll. Zhou Xin, 3 males (NAU); Du-jiang-yan County, Qing-cheng Hou-shan Scenic Area, Tong-ling-gou Stream, 600 m upstream of Qing-quan-yuan Hotel, 8.7 km S main gate, 30.9301°N, 103.4957°E, 985 m elevation, 10-vii-2005, Coll. Sun Chang-hai, 1 male (NAU).

#### Diagnosis.

The male of this species is similar to that of *Nyctiophylax (Paranyctiophylax) sagax* Mey, 1995 in the thick base and slender finger-shaped distal part of each inferior appendage and in the shape of the preanal appendages. However, the 2 species differ in that the mesoventral process of each preanal appendage is evenly curved caudoventrad and blunt apically, and there is no internal spine at the apex of the phallus in the new species. In contrast, in *Nyctiophylax sagax*, the mesoventral process of each preanal appendage is curved ventrad at a right angle in the middle and acute apically, and there is a longitudinal row of internal spines at the apex of the phallus.

#### Description of adult male.

Head light brown with yellowish antennae, pronotum yellowish, meso- and metanota brown, with warts and thoracic legs yellowish, forewings light brown. Length of body with folded forewings: 5.5–7.0 mm. (n=10).

**Figure 6.**
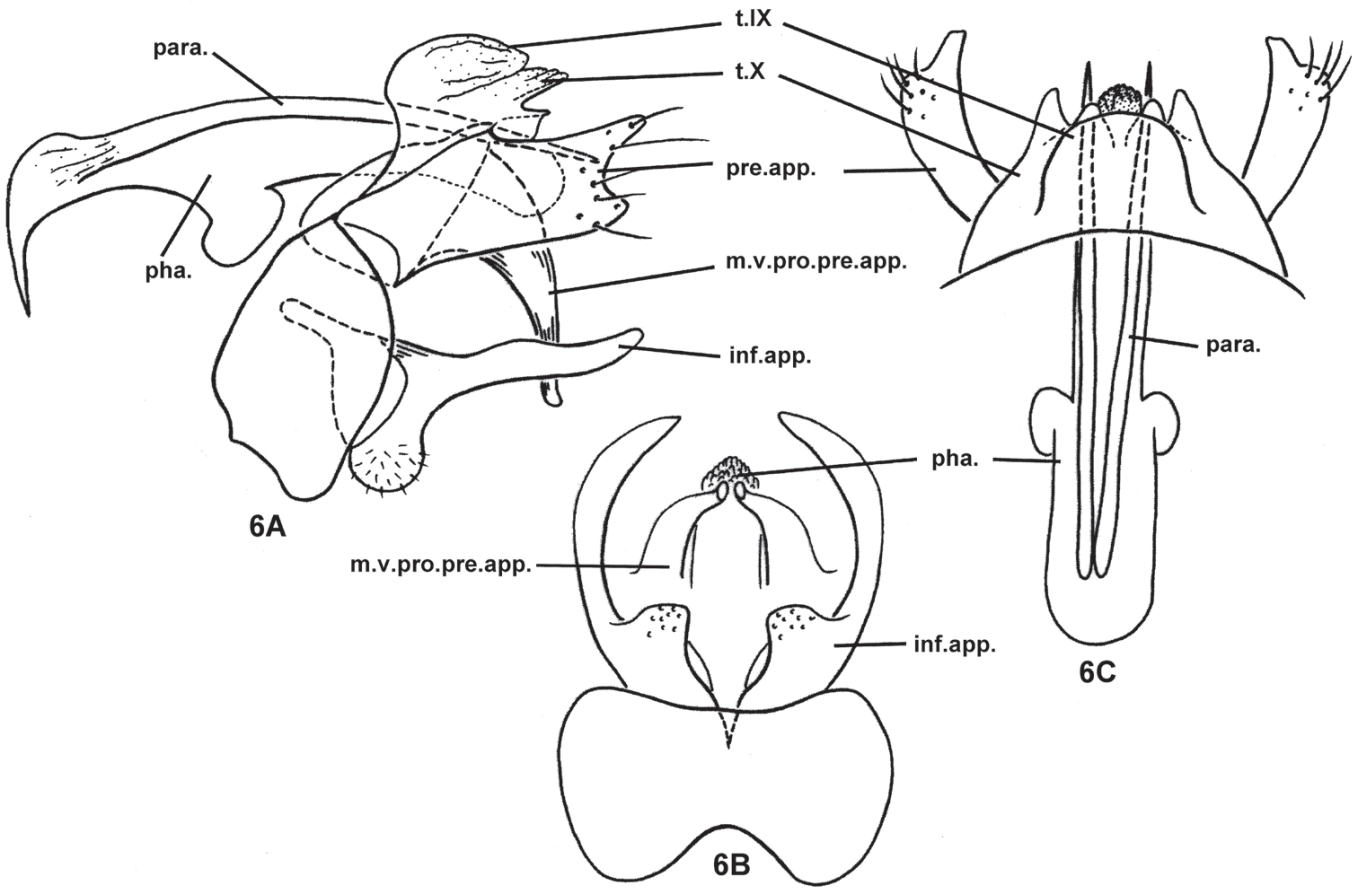
*Nyctiophylax (Paranyctiophylax) gracilis* Morse, Zhong & Yang, sp. n., male genitalia. **6A** left lateral view **6B** ventral view **6C** dorsal view. inf.app. = inferior appendage; m.v.pro.pre.app. = mesoventral process of a preanal appendage; para. = paramere; pha. = phallus; pre.app. = preanal appendage; s.IX = sternum IX; t.IX = tergum IX; t.X = tergum X.

Male genitalia. Tergum IX ovate in lateral view (Fig. 6A) and subquadrate in dorsal view (Fig. 6C), completely membranous. In lateral view (Fig. 6A), sternum IX (s.IX) quadrate, in ventral view (Fig. 6B), posteromesal margin slightly concave, anteromesal margin with shallow, “V–shaped” excisionTergum X broad, widely divided apically, each process with small lobe apicoventrally, acute in lateral view and blunt in dorsal view. Preanal appendages rectangular in lateral view (Fig. 6A), about 2 times as long as wide, each with broad incision apically; mesoventral processes evenly curved caudoventrad, broad at bases, each gradually reduced to a narrow, blunt apex; in ventral view (Fig. 6B) apices extended mesad beneath phallus. Inferior appendages each with basoventral part much thicker than slender, digitate, incurved distal part; in ventral view (Fig. 6B) basoventral parts each produced in subquadrate lobe with truncate apex. Phallus with phallobase broad and depressed, pair of slender parameres slightly longer than phallicata; phallicata slender, round at apex (Figs 6B, 6A, 6C).

#### Female and immature stages.

Unknown.

#### Distribution.

Oriental Biogeographic Region, China (Jiang-xi, Zhe-jiang, An-hui, Si-chuan).

#### Etymology.

*Gracilis*, Greek adjective, „slender,“ referring to the slender distal part of each inferior appendage.

### Nyctiophylax (Paranyctiophylax) pungens

Morse, Zhong & Yang sp. n.

urn:lsid:zoobank.org:act:D6571186-7FBE-420E-BB82-FA638FD302D1

http://species-id.net/wiki/Nyctiophylax_pungens

Fig. 7

Nyctiophylax (Paranyctiophylax) pungens Li 1998: 93–94, figs 4.19–4.21, *nomen nudum*.

#### Type material.

Holotype male, PRC, An-hui Province, Jin County, Song Village, Ding-xi Stream, 33 km E of Jin County, 08-vi-1990, 120 m elevation, coll. JC Morse, Sun Chang-hai, and Yang Lian-fang, deposited in NAU.

#### Paratypes.

PRC, An-hui Province, 3 males, same data as holotype (NAU); 20 males, Qi-men County, 29.8°N, 117.7°E, Peng-long Township, Yang Village, provincial highway at 122 km marker, 249 m elevation, 04-vi-2003, Shan Lin-na, Lu Shuang (NAU); 5 males, Peng-long Township, Yang Village, provincial highway at 122 km marker, 29-v-2002, coll. Shan Lin-na, Hu Ben-jin (NAU); 1 male, Peng-long Township, Yang Village provincial highway at 122 km marker, 25-viii-2002, coll. Hu Ben-jin and Lu Shuang (NAU).

PRC, Jiang-xi Province: 1 male, Mt. Wu-yi National Nature Preserve, Li-tou-jian Stream, 100 m upstream of protected area marker, 27.9862°N, 117.8561°E, 342 m elevation, 05-vi-2005, Coll. Yang Lian-fang, CJ Geraci (NAU); 1 male, Mt. Wu-yi National Nature Preserve, Li-tou-jian Stream, 500–900 m upstream of protected area marker, 27.9803°N, 117.8619°E, 375–404 m elevation, 05-vi-2005, Coll. Sun Chang-hai,, Zhou Chang-fa, Zhou Xin (NAU); 1 male, Mt. Wu-yi National Nature Preserve, Lei- gu-ling Stream, 27.9914°N, 117.8911°E, 424 m elevation, 04-vi-2005, Coll. Yang Lian-fang, CJ Geraci (NAU); 12 males, Mt. Wu-yi National Nature Preserve, Lei-gu-ling Stream, 28.0045°N, 117.8814°E, 344 m elevation, 04-vi-2005, Coll. Zhou Xin, Zhou Chang-fa (NAU); 3 males, Mt. Wu-yi National Nature Preserve, Lei-gu-ling Stream, 04-vi-2005, Coll. Sun Chang-hai (NAU).

#### Diagnosis.

The genitalia of this species are very similar to those of *Nyctiophylax (Paranyctiophylax) nahum* (Malicky & Chantaramongkol, 1993) in the long basoventral process of each inferior appendage and in the long slender dorsal process of each preanal appendage. However, in the new species the dorsal process of each preanal appendage is long and stout, curved caudolaterad in dorsal view, but straight and much more slender in *Nyctiophylax nahum*; and the basoventral process of each inferior appendage forms a 45-degree angle with the main body of the appendage in lateral view in the new species, but an angle of about 90 degrees in *Nyctiophylax nahum*. The shape of the mesoventral process of each preanal appendage is somewhat similar to that of *Nyctiophylax (Paranyctiophylax) devanampriya* (Schmid, 1958), but in that species the basoventral process of each inferior appendage is much shorter than that of the new species, which is about 1/3rd as long as the main body of the appendage.

#### Description of adult male.

Head grayish brown with yellowish antennae, pronotum light gray, meso- and metanota brown dorsally, yellowish lateroventrally with concolorous thoracic legs, forewings light brown. Length of body with folded forewings: 4.3–5.5 mm (n=15).

**Figure 7.**
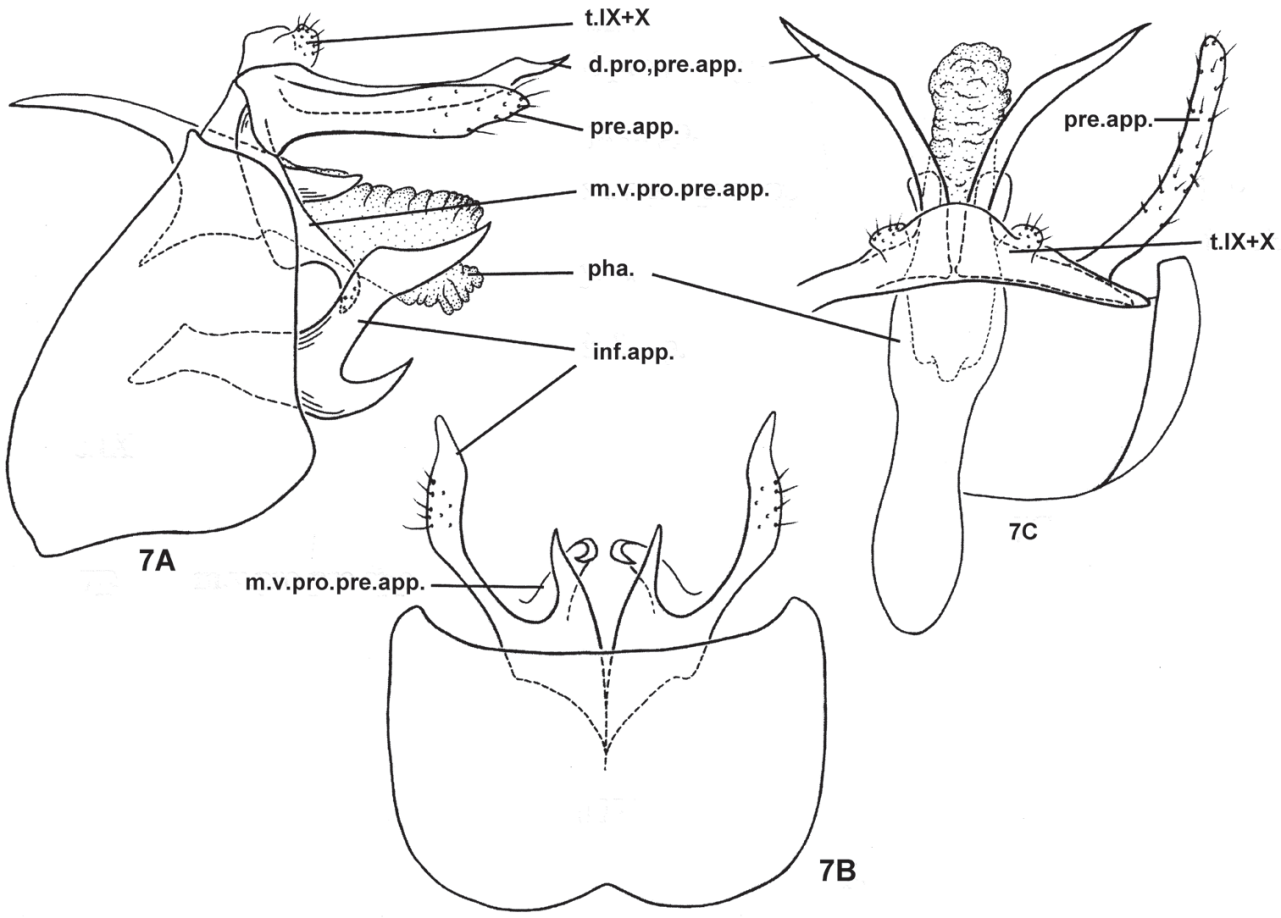
*Nyctiophylax (Paranyctiophylax) pungens* Morse, Zhong & Yang, sp. n., male genitalia. **7A** left lateral view **7B** ventral view **7C** dorsal view. d.pro.pre.app. = dorsal process of a preanal appendage; inf.app. = inferior appendage; m.v.pro.pre.app. = mesoventral process of a preanal appendage; pha. = phallus; pre.app. = preanal appendage; s.IX = sternum IX; t.IX = tergum IX; t.X = tergum X.

Male genitalia. Tergum IX short, membranous, broadly rounded apically in dorsal view (Fig. 7C). Sternum IX relatively large, in lateral view (Fig. 7A) subtriangular, with short posterodorsal margins 1/3rd as long as ventral surface, anterolateral margins oblique and convex, especially subventrally, posterolateral margins nearly straight, vertical; in ventral view (Fig. 7B), posteromesal margin broadly and shallowly excised, anteromesal margin with short V-shaped excision. Tergum X fused laterally to tergum IX, represented as 2 small setose lateral lobes. Without obvious intermediate appendages. Preanal appendages slender, straight in lateral view (Fig. 7A), curved slightly caudad in dorsal view (Fig. 7C); each with long dorsal process curved caudolaterad, acute apically; its mesoventral process broad at basal 2/3rds, with acute apex hooked slightly ventrad and mesad beneath phallus. Inferior appendages each with acute posterior basoventral process half as long as main body of appendage; main body of appendage slender, setose, and acute apically. Phallus with phallobase sclerotized, broader apically, with pair of short sclerites dorsally, phallicata membranous, without internal spines.

#### Female and immature stages.

Unknown.

#### Distribution.

Oriental Biogeographic Region, China (An-hui, Jiang-xi).

#### Etymology.

*Pungens*, Latin adjective, „acute,“ referring to the acute, posterior basoventral process of each inferior appendage.

### Nyctiophylax (Paranyctiophylax) auriculatus

Morse, Zhong & Yang sp. n.

urn:lsid:zoobank.org:act:5B0B4469-C51F-46C3-B286-7AE27A5244E9

http://species-id.net/wiki/Nyctiophylax_auriculatus

Fig. 8

Nyctiophylax (Paranyctiophylax) auriculatus Li 1998: 94–96, figs 4.22–4.24, *nomen nudum*.

#### Type material.

Holotype male, PRC, Jiang-xi Province, Wu-yuan County, Qin-hua River, 57 km N of Wu-yuan, 29.15°N, 117.53°E, 25-v-1990, 250 m elevation, collected by JC Morse, Yang Lian-fang, and Sun Chang-fai, deposited in NAU.

#### Paratype.

PRC, Guang-dong Province: Bo-luo County, Mt. Luo-fu, unnamed stream, 400 m on trail to Shan-bei-shui, trailhead 3.2 km W of ridge of Mt. Cha, 23.3190°N, 114.0115°E, 290 m elevation, 01-vi-2004, Coll. JC Morse, Zhou Xin, CJ Geraci, 1 male (NAU).

#### Diagnosis.

The genitalia of the new species are similar to those of *Nyctiophylax (Paranyctiophylax) hjangsanchonus* (Botosaneanu, 1970) and *Nyctiophylax (Paranyctiophylax) cascadensis* (Malicky, 1995) in the short and broad preanal appendages and in the pair of long and slender phallic parameres. However, in lateral view, where the preanal appendages are semicircular in *Nyctiophylax hiangsuchonus* and triangular in *Nyctiophylax cascadensis*, they are somewhat quadrate in the new species. Also, the inferior appendages each have a mesal process in *Nyctiophylax hiangsuchonus*, a ventral process in *Nyctiophylax cascadensis*, and no conspicuous process in the new species.

#### Description of adult male.

Head and thorax light yellowish brown with yellowish antennae, pronotum light brown, meso- and metanota yellowsh-brown dorsally, pale yellowish lateroventrally with concolorous thoracic legs, forewings light brown. Length of body with folded forewings: 5.6–5.8 mm (n=2).

**Figure 8.**
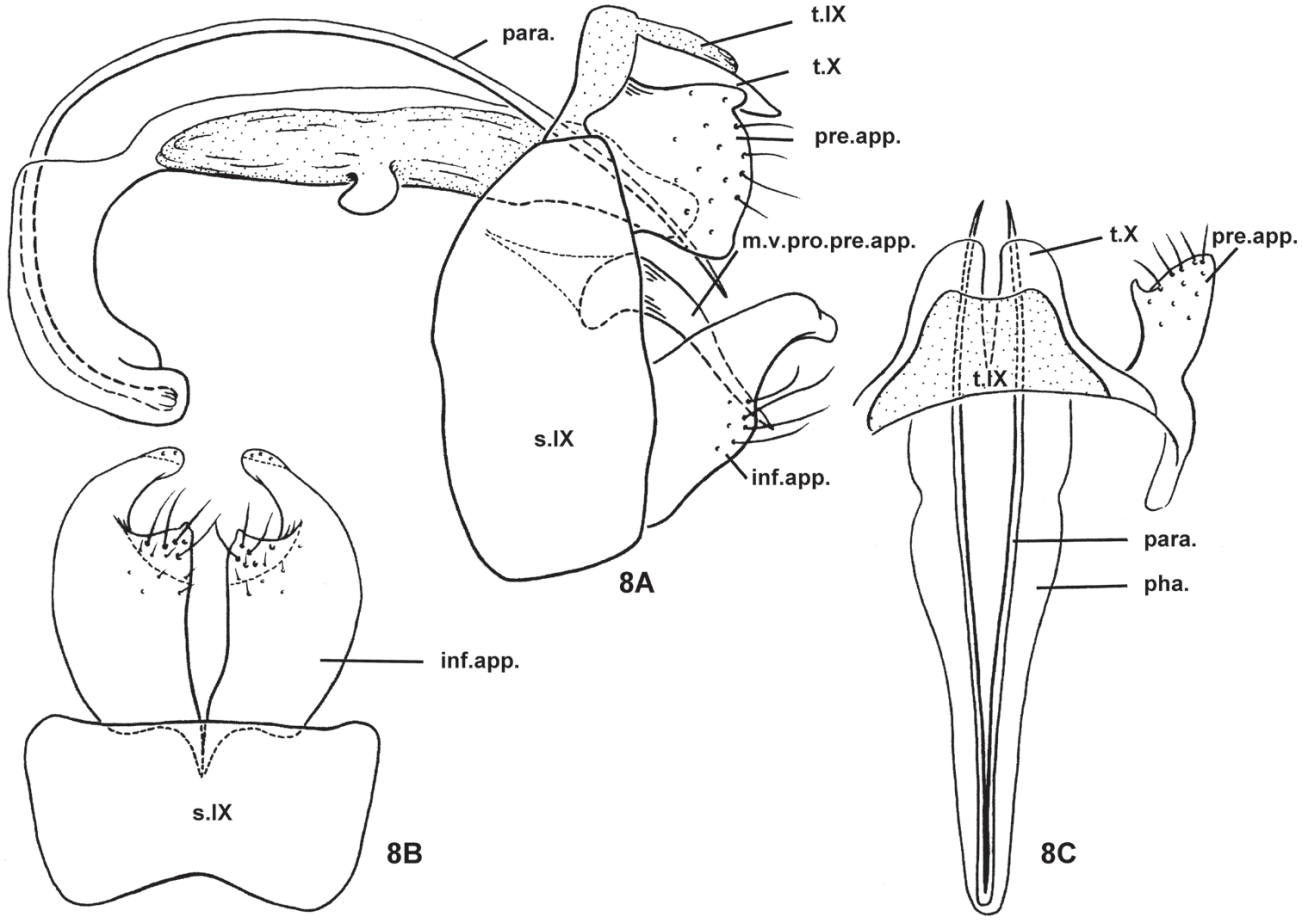
*Nyctiophylax (Paranyctiophylax) auriculatus* Li & Yang, sp. n., male genitalia. **8A** left lateral view **8B** ventral view **8C** dorsal view. inf.app. = inferior appendage; m.v.pro.pre.app. = mesoventral process of a preanal appendage; para. = paramere; pha. = phallus; pre.app. = preanal appendage; s.IX = sternum IX; t.IX = tergum IX; t.X = tergum X.

Male genitalia. Tergum IX membranous, short, trapezoid in dorsal view (Fig. 8C), with shallow incision on posterior margin. In lateral view (Fig. 8A), sternum IX tall, subquadrangular, with anterolateral margins convex, dorsal margins short and round, posterolateral margins almost straight and vertical, ventral surface about twice as long as dorsal margin; in ventral view (Fig. 8B), posterior margin nearly straight, anteromesal margin with broad and shallow excision. Tergum X lightly sclerotized, deeply divided apicomesally into 2 broad lobes in dorsal view (Fig. 8C). Without obvious intermediate appendages. Preanal appendages shorter than tergum X, almost square in lateral view (Fig. 8A), obliquely truncate; with long, stout mesoventral process (m.v.pro.pre.app.) tapering gradually to acute apex directed caudoventrad. Inferior appendages each with basal 2/3rds much broader than apical 1/3rd; basoventral process not visible in lateral view; distal 1/3rd narrower and curved mesad with obtuse apex in ventral and lateral views (Figs 8B, 8A). Phallus with sclerotized phallobase twice as long as phallicata, curved ventrad and tapering anteriorly and with pair of short blunt lobes posteroventrally; parameres (para.) longer than phallus, arising at ventral, anterior end of phallobase; phallicata membranous dorsally, without internal spines.

#### Female and immature stages.

Unknown.

#### Distribution.

Oriental Biogeographic Region, China (Jiang-xi, Guang-dong).

#### Etymology.

*Auriculatus*, Latin adjective, “ear-like,” referring to the shape of the preanal appendages in lateral view.

## Supplementary Material

XML Treatment for Plectrocnemia verticalis

XML Treatment for Plectrocnemia acuminata

XML Treatment for Plectrocnemia cryptoparamere

XML Treatment for Plectrocnemia qianshanensis

XML Treatment for Nyctiophylax (Nyctiophylax) senticosus

XML Treatment for Nyctiophylax (Paranyctiophylax) gracilis

XML Treatment for Nyctiophylax (Paranyctiophylax) pungens

XML Treatment for Nyctiophylax (Paranyctiophylax) auriculatus
